# Limitations of Bedside Lung Ultrasound in Neonatal Lung Diseases

**DOI:** 10.3389/fped.2022.855958

**Published:** 2022-04-26

**Authors:** Xiaolei Liu, Shuyu Si, Yiyi Guo, Hui Wu

**Affiliations:** Department of Neonatology, The First Hospital of Jilin University, Changchun, China

**Keywords:** lung ultrasound, congenital pulmonary airway malformation, pulmonary bullae, pneumothorax, pulmonary interstitial emphysema, pneumonia

## Abstract

Lung ultrasound is a technique that has rapidly developed in recent years. It is a low-cost, radiation-free, and easy-to-operate tool that can be repeatedly performed at the bedside. Compared to chest X-ray, lung ultrasound has high sensitivity and specificity in the diagnosis of neonatal respiratory distress syndrome, transient tachypnoea of newborns and pneumothorax. Lung ultrasound has been widely used in neonatal intensive care units. However, due to the physical barriers of air, where ultrasonic waves cannot pass and therefore reflection artifacts occur, it has limitations in some other lung diseases and cannot fully substitute for chest X-rays or CT/MRI scanning. This review describes these limitations in detail and highlights that if clinical symptoms are not effectively alleviated after medical treatment or the clinical presentation is not compatible with the ultrasound appearances, then chest X-rays or CT/MRI scanning should be performed to avoid misdiagnosis and mistreatment.

## Introduction

Lung ultrasound originated in adult critical care medicine. In 1990, Avni et al. (1) first reported that lung ultrasound might be an alternative to chest radiography for the diagnosis of neonatal respiratory distress syndrome. Since then, neonatal and pediatric lung ultrasound has also developed rapidly. The ultrasound appearances of neonatal respiratory distress syndrome (NRDS) (2), transient tachypnoea of newborns (TTN) (3), neonatal pneumonia (4), pneumothorax (5) and meconium aspiration syndrome (MAS) (6) have been clearly described. It was found that lung ultrasound had high specificity, sensitivity, positive predictive value and negative predictive value and showed high consistency with chest X-ray in detecting the above disorders (7). International evidence-based guidelines on point-of-care ultrasound (POCUS) for critically ill neonates and children were issued by the POCUS Working Group of the European Society of Pediatric and Neonatal Intensive Care in 2020 (8). Given that bedside lung ultrasound is more sensitive to tiny foci close to the pleura than chest radiography is and that lung ultrasound is a low-cost, radiation-free, and easy-to-operate tool that can be repeatedly performed at the bedside, lung ultrasound has been widely used in neonatal intensive care units (NICUs). In recent years, lung ultrasound has become the preferred examination for NRDS, TTN, neonatal pneumonia, pneumothorax and MAS instead of chest X-ray in some neonatal departments (9). Every neonatologist who had been trained by two professional ultrasound physicians for 3 months has grasped the essential operational skill sets, and interpretation of ultrasound images by training. Bedside lung ultrasound has been widely used in our NICU in the past 2 years, decreasing bedside chest X-ray by about 50%.

However, according to the application of lung ultrasound, reading of the relevant literature, and problems encountered in actual clinical practice, bedside lung ultrasound also has limitations in some other lung diseases that much less frequently occur in neonatal period, and cannot fully substitute for chest X-rays or CT (computer tomography) or MRI (magnetic resonance imaging) which can monitor early lung disease in neonates and delineate fine intrapulmonary airways and vasculature. The limitations are summarized as follows.

## Limitations of Lung Ultrasound

### Congenital Pulmonary Airway Malformation

Congenital pulmonary airway malformations (CPAMs) are polycystic immature alveolar tissues formed by abnormal branching development of the lung in *utero*, disruption of the lung architecture and excessive growth of terminal bronchioles. Because the fetal lung is filled with fluid, lesions can be detected by perinatal ultrasound examination. The number of CPAMs detected in *utero* is gradually increasing with the promotion of perinatal ultrasound. Neonatal lung ultrasound is limited in that it misses lesions because of air-filled lungs. Hence, lung CT examination is required for postnatal diagnosis. The ultrasound appearances of CPAM after birth have also been gradually studied with the development of lung ultrasound. Dietrich et al. (10) and Quercia et al. (11) reported the ultrasound image of CPAM showed a single large cystic lesion or multiple hypoechoic lesions which were located in subpleural region of the lung. Yousef et al. (12) described the ultrasonic manifestations of CPAM, such as a single large cystic lesion, multiple tiny cysts, and/or irregular consolidation. However, a single large cyst can also be found in certain congenital pulmonary cyst cases, which cannot be differentiated by lung ultrasound. Merli et al. (13) reported a case of prenatal suspected CPAM. It presented multiple cystic lesions, of which the largest lesion was found to be distant from the pleura by CT scan, while postnatal lung ultrasound examination only revealed subpleural consolidation. The reason was that the alveoli around the lesions were normally inflated, and total reflection was formed between the normal alveoli and the probe. Thus, lesions away from the pleura could not be clearly observed, and normal or slightly abnormal sonograms were mainly yielded by lung ultrasound. So lung ultrasound is possible to describe CPAM, but has limitations in lesions distant to pleura. Though it is at least possible to reduce X-rays in good clinical presentations, MRI / CT is still needed for further assessment.

### Pulmonary Bullae and Subcutaneous Emphysema

Two articles (14, 15) published in 2019 indicated that lung sliding could differentiate bullae and pneumothorax which could not differentiate on chest x-ray, but giant bullae cannot be visualized using ultrasound. The giant bullae mimic a normal lung but the hint is the clinical presentation, that is not in line with the normal lung findings. When the probe emits an acoustic beam to perforate through the tissues and organs, physical phenomena such as reflection, refraction, and scattering occur. The penetration of acoustic beams is related to the components of tissues and organs. When the acoustic beam encounters gas, it will be totally reflected. Certain lesions cannot be found by lung ultrasound due to the influence of gas in front of the lesion. When pulmonary bullae form, lung ultrasound cannot show the presence of pulmonary bullae due to the total reflection of the acoustic beam caused by a large amount of gas in the bubble. Similarly, subcutaneous emphysema may also affect the results of lung ultrasound due to the same mechanism described earlier where there is a large amount of gas interfering with the acoustic beam. This was confirmed by two letters (16, 17) which stated that subcutaneous emphysema limited the utility of ultrasound. So, chest x-ray or CT should be performed to assist diagnosis.

### The Volume of the Pneumothorax

Although lung ultrasound is sensitive for the diagnosis of pneumothorax, it is unable to measure the volume of the pneumothorax. Liu et al. (18) conducted a study to explore the accuracy and reliability of ultrasonography for the diagnosis of neonatal pneumothorax and found that the main ultrasonographic signs of pneumothorax included disappearance of lung sliding, presence of a pleural line and A-line, presence of lung points in children with mild to moderate pneumothorax, absence of lung points in children with severe pneumothorax, absence of a B-line and lung consolidation in the area of pneumothorax. The sensitivity, specificity, positive predictive value and negative predictive value of lung sliding disappearance and the presence of pleural lines and A-lines in diagnosing pneumothorax were 100%. Raimondi et al. (5) reported that lung ultrasound had high accuracy in detecting pneumothorax, was superior to clinical evaluation and shortened the time of diagnosis and treatment. International evidence-based guidelines also indicated that lung ultrasound was helpful to accurately detect pneumothorax in neonates and children (Grade B) (8). It is worth noting that the absence of lung sliding was also observed in other disorders, such as acute respiratory distress syndrome, pulmonary atelectasis, pneumonia, decreased lung compliance, and foreign body aspiration (19–22). As a result, the diagnosis of pneumothorax cannot rely on the disappearance of lung sliding alone, but the presence of lung sliding can exclude pneumothorax. Another conditions, that could help to differentiate the importance of a tension pneumothorax are mid-line shifting of the heart and thymus, which could be evaluated by experienced ultrasound users. In addition, the usefulness of lung ultrasound in the determination of pneumothorax size was not certain. Liu et al. (18) found that lung points existed in children with mild to moderate pneumothorax and disappeared in children with severe pneumothorax. Volpicelli et al. (23) proposed that lung ultrasound could quantify the pneumothorax volume by locating lung points on the chest wall, and the lateral progression of lung points on the chest wall corresponded to an increase in the extent of pneumothorax. However, the thickness of pneumothorax cannot be measured due to the total reflection caused by the gas, and the usefulness of lung ultrasound for quantifying the pneumothorax size needs further validation in practice. Anyway, ultrasound findings have to be compared to the clinical presentation and other findings, like oxygenation level. We suggest the quantification of the pneumothorax is evaluated by chest X-ray and CT.

### Pulmonary Interstitial Emphysema

Pulmonary interstitial emphysema cannot be diagnosed by lung ultrasound. One case study reported (24) the role of lung ultrasound in the follow-up of localized interstitial emphysema. The infant again presented tachypnoea after continuous positive airway pressure for 72 h. Lung ultrasound revealed a normal pattern of the right lung and non-coalescent B-lines on the left side, while thoracic computed tomography showed localized interstitial emphysema of the left upper lobe. This ultrasonic finding (non-coalescent B-line) was observed in other lung diseases and was not specific for emphysema. In patients with emphysema, we speculate that lung ultrasound often showed normal sonographic manifestations with the presence of lung sliding and an A-line due to decreased elastance of airway distal to terminal bronchioles, overinflation of the lung, increases in lung volume, and a widened intercostal space. Therefore, the ultrasound appearance of pulmonary interstitial emphysema needs further research and CT or chest X-ray can be used to diagnose pulmonary interstitial emphysema.

### Pathogenesis of Pneumonia

The diagnosis of pneumonia by lung ultrasound should be combined with the medical history. A distinction between infected and noninfected consolidation cannot be made on lung ultrasound. The ultrasound images of pneumonia include the presence of pulmonary consolidation with irregular margins and dynamic air bronchograms, pleural effusion, pleural line abnormalities or the absence of lung sliding, and alveolar–interstitial patterns in the adjacent areas (25). Since lobar pneumonia occurs in lung segments and lobes, and the sensitivity of ultrasonography for diagnosing lobar pneumonia is high, the sonographic appearances of lobar pneumonia include large consolidation with dynamic air bronchograms and/or pleural effusions. It should be noted that lobar pneumonia plays a minor role in neonatal pneumonia, so specific findings help to differentiate in respiratory distress in children but cannot be very useful to help diagnosing a pneumonia in neonates. It was reported that large lung consolidation with irregular margins had a sensitivity of 100% and specificity of 100% for the diagnosis of neonatal pneumonia compared to neonates without a lung diseases (26). Of note, the detection of consolidation by lung ultrasound is associated with the size of the consolidation and the distance between the consolidation and the pleural surface. The smaller consolidations far away from the pleura and areas under the bony structure may be missed by lung ultrasound. Large lung consolidations can be observed in NRDS, acute respiratory distress syndrome and MAS; small consolidations can be observed in NRDS characterized by progressive dyspnoea shortly after birth and bronchopulmonary dysplasia with long-term dependence on oxygen, and thus, a medical history is crucial for the differential diagnosis. Additionally, although a distinction between a small consolidation >1 cm and 1 <1 cm had been studied, the diagnostic threshold of the consolidation size for pneumonia was not presented explicitly in the study (27, 28). It was reported in the literature (29) that viral pneumonia presented with subpleural small consolidation (diameter<1 cm) and/or a B-line and that bacterial pneumonia presented with lung consolidation with dynamic air bronchograms. We argued that the cut-off value of a consolidation diameter <1 cm for distinguishing between bacterial and viral infections was controversial, although a small consolidation with a diameter <1 cm was undetectable by chest radiography. Another study (30) reported LUS findings of viral pneumonia and sepsis-related respiratory failure, both groups showed consolidations with air bronchograms, pleura line abnormalities, B-pattern, lung pulse, pleural effusion and disappearance of lung sliding sign. Several studies (31, 32) reported that acute bronchiolitis presented with irregular pleural lines, subpleural consolidation and interstitial syndrome, which was similar to viral pneumonia. A study (28) evaluated the diagnostic accuracy of lung ultrasound for the detection of pneumonia in hospitalized children with acute bronchiolitis, reporting that 10 cases showed false-positive ultrasonic findings and that 9 of the cases consisted of subcentimetric pneumonia. Therefore, the underlying limitation in neonatal lung ultrasound is the problem of the differentiation of neonatal respiratory diseases like RDS, TTN, MAS or neonatal pneumonia. On the other hand, even the chest X-ray is not perfect in the differentiation of these diseases and clinical data is still mandatory. For neonates with small subpleural consolidations, lung ultrasound is not useful to distinguish between bacteria or viral pneumonia or acute bronchiolitis. The differential diagnosis requires a combination of clinical examination and medical history.

## Conclusion

Due to the limitations of lung ultrasound, we believe that lung ultrasound cannot fully substitute for chest X-ray. CT or MRI should be used to evaluate CPAM distant to pleura. Chest X-ray or CT should be recommended to diagnose pulmonary bullae, subcutaneous emphysema, pulmonary interstitial emphysema and quantify the volume of the pneumothorax. If the clinical symptoms are not effectively alleviated after medical treatment or the clinical presentation is not compatible with the ultrasound appearances, then further radiological interventions should be used to avoid misdiagnosis and mistreatment.

## Author Contributions

XL wrote the first draft and edited the final manuscript. SS was involved in the conceptual framing of the manuscript. YG was involved in the review of background literature. HW devised the concept of the review, edited the final version and approved the overall article. All authors contributed to the article and approved the submitted version.

## Funding

The review is funded by grants from the Scientific Research Foundation of the department of science and technology of Jilin Province, China (Grant Number: 20190701050GH).

## Conflict of Interest

The authors declare that the research was conducted in the absence of any commercial or financial relationships that could be construed as a potential conflict of interest.

## Publisher's Note

All claims expressed in this article are solely those of the authors and do not necessarily represent those of their affiliated organizations, or those of the publisher, the editors and the reviewers. Any product that may be evaluated in this article, or claim that may be made by its manufacturer, is not guaranteed or endorsed by the publisher.

## Abbreviations

NRDS: neonatal respiratory distress syndrome
TTN: transient tachypnoea of newborns
MAS: meconium aspiration syndrome
POCUS: point-of-care ultrasound
NICUs: neonatal intensive care units
CPAMs: congenital pulmonary airway malformations
CT: computer tomography
MRI: magnetic resonance imaging.
